# Genome-Resolved Metagenomics of the Chicken Gut Microbiome

**DOI:** 10.3389/fmicb.2021.726923

**Published:** 2021-08-16

**Authors:** Maia Segura-Wang, Nikolaus Grabner, Andreas Koestelbauer, Viviana Klose, Mahdi Ghanbari

**Affiliations:** BIOMIN Research Center, Tulln, Austria

**Keywords:** chicken, gut microbiome, metagenomics, MAG, CAZymes

## Abstract

Increasing evidence shows that the chicken gastrointestinal microbiota has a major effect on the modulation of metabolic functions and is correlated with economic parameters, such as feed efficiency and health. Some of these effects derive from the capacity of the chicken to digest carbohydrates and produce energy-rich metabolites such as short-chain fatty acids (SCFA) and from host-microbe interactions. In this study, we utilized information from metagenomic assembled genomes (MAGs) from chicken gastrointestinal tract (GIT) samples, with detailed annotation of carbohydrate-active enzymes (CAZymes) and genes involved in SCFA production, to better understand metabolic potential at different ages. Metagenomic sequencing of 751 chicken GIT samples was performed to reconstruct 155 MAGs, representing species which belong to six phyla, primarily Firmicutes followed by Proteobacteria. MAG diversity significantly (*p* < 0.001) increased with age, with early domination of Lachnospiraceae, followed by other families including Oscillospiraceae. Age-dependent shifts were observed in the abundance of genes involved in CAZyme and SCFA production, exemplified by a significant increase in glycosyltransferases (GTs) and propionic acid production pathways (*p* < 0.05), and a lower abundance of glycoside hydrolases (GHs) (*p* < 0.01). Co-occurrence analysis revealed a large cluster highly interconnected by enzymes from GT2_2 and GH3 families, underscoring their importance in the community. Furthermore, several species were identified as interaction hubs, elucidating associations of key microbes and enzymes that more likely drive temporal changes in the chicken gut microbiota, and providing further insights into the structure of the complex microbial community. This study extends prior efforts on the characterization of the chicken GIT microbiome at the taxonomic and functional levels and lays an important foundation toward better understanding the broiler chicken gut microbiome helping in the identification of modulation opportunities to increase animal health and performance.

## Introduction

There is a growing interest in animal microbiome research because the gastrointestinal microbiota modulates several important physiological functions, such as digestion and absorption, energy metabolism, and immune system development, and help in the prevention of infections (Stanley et al., 2012; Deusch et al., 2015; Brugman et al., 2018; Wen et al., 2019). In chickens, alterations in the gastrointestinal tract (GIT) microbial community are correlated with several economically important traits, such as feed efficiency, body weight, abdominal fat mass, and health (Kohl, 2012; Yan et al., 2017; Broom and Kogut, 2018; Huang et al., 2018; Shang et al., 2018; Xiong et al., 2018; Kubasova et al., 2019; Ocejo et al., 2019; Shah et al., 2019; Wen et al., 2019). Indeed, it has been shown that by using numerous carbohydrate-active enzymes (CAZymes), members of the chicken gut microbiota are able to breakdown plant-derived fibers, degradation of dietary carbohydrates and host-derived glycans (El Kaoutari et al., 2013), thereby producing organic acids such as short-chain fatty acids (SCFAs) that play crucial roles in energy metabolism, gastrointestinal physiology, and immune function (Józefiak et al., 2004; Koh et al., 2016).

Furthermore, the composition of the gut microbiome is known to change with the host age (Yatsunenko et al., 2012; Falony et al., 2016; Stewart et al., 2018). Studies based on human and mice data have shown that the aging process has a strong influence on the distribution of taxonomic groups and functional capacity of the GIT bacterial community (Yatsunenko et al., 2012; Langille et al., 2014; Stewart et al., 2018). In the broiler chicken some reports exist about the compositional changes of the microbiome, due to successional replacement and the colonization of more stable taxa as the birds advance in age (Lu et al., 2003; Mohd Shaufi et al., 2015; Huang et al., 2018). Since these alterations have been linked to the health status of the hosts (Claesson et al., 2012; Awad et al., 2016), it is important to better understand the shifts in specific bacterial groups and the functional capacity of these organisms to generate better links to performance and health.

During the last decade, high-throughput sequencing technologies have extensively facilitated microbial community studies to explore taxonomic and functional diversity in the livestock microbiome, including broiler chickens (Sergeant et al., 2014; Deusch et al., 2015; Huang et al., 2018). For instance, a set of 469 metagenomic assembled genomes (MAGs) from the chicken cecum was described by Glendinning et al. (2020) and more recently a large collection of 5,595 MAGs representing 853 metagenomic species were described (Gilroy et al., 2021). The former paper found differences in the microbial composition depending on the chicken line and diet, while the latter focused on a thorough taxonomic classification of the constructed MAGs, but not on their functional potential. Yet, there is still a paucity of data regarding the functional annotation of chicken gut metagenome and their temporal variability such as the ones involve in coding CAZymes and SCFA production which play crucial nutritional roles for the host (Józefiak et al., 2004; Koh et al., 2016). It is also important to identify highly interconnected key enzymes and microbes because they which are more likely to drive changes in the microbiome.

In this study, we present a comprehensive genome-resolved analysis of 751 chicken gut metagenomes, resulting in over 150 metagenomic assembled bacterial genomes. Furthermore, we conducted an integrative analysis of the complex interplay that occurs in the GIT between microbes with different set of the genes encoding CAZymes and SCFAs. Our results expand the knowledgebase of the mechanisms governing changes in the gut microbiome and underscore the need for further studies on the structure and function of the chicken gut microbiome.

## Materials and Methods

### Animals and Metagenomic Samples

All experiments described in this study were conducted at the Center of Animal Nutrition (Tulln, Austria) with the approval of the Lower Austrian Region Government, Group of Agriculture and Forestry, Department of Agricultural Law (approval code LF1-TVG-57/005-2018), and following the European Guidelines for the Care and Use of Animals for Research Purposes (European Council, 2010).

Two hundred and forty healthy-looking 1-day-old male broiler chickens (Ross 308), with similar body weight of around 55 g, were selected for this experiment and divided into 24 slatted floor pens with *ad libitum* access to water and standard diet feed. The feed consisted of a standard diet mainly composed of cornmeal (53%) and soybean meal (31%) during the first 15 days and was changed to cornmeal (60%) and soybean meal (25%) from day 15 to day 35. The animals did not receive coccidiostats nor any vaccinations. Two birds per cage were randomly selected at defined time points (age 3, 14, 21, and 35 days), and samples were taken from ileum, ceca, and colon. In all these gut sections, mucosa and digesta were collected. The animals were euthanized by asphyxiation with CO_2_. All materials were stored in nucleic acid preservation buffer (NAP) (Camacho-Sanchez et al., 2013) at 4°C until further processing.

### Library Preparation and Sequencing

DNA was extracted from the collected samples using the QIAamp PowerFecal DNA Kit (Qiagen) following the manufacturer’s instructions. Library preparation and sequencing were performed by LGC Genomics GmbH, using 150 bp paired-end reads (Illumina NextSeq 500 V2).

### Genome Assembly, Quality, and Abundance

The Illumina sequencing reads were demultiplexed, and adaptors were trimmed using trimmomatic (version 0.38) (Bolger et al., 2014). The removal of host and human sequencing reads was performed using DeconSeq (version 0.4.3) (Schmieder and Edwards, 2011) to increase the proportion of microbial sequences. The decontaminated reads were used to generate MAGs by means of a combination of single-sample assemblies and a co-assembly using the sequencing reads of all metagenomic samples, as described in previous studies (Stewart et al., 2019; Glendinning et al., 2020). Individual samples were assembled using IDBA-UD (Peng et al., 2012). Additionally, all the sequencing data generated from different samples was pooled and used for co-assembly utilizing the ultra-fast and memory-efficient (meta-)genome assembler MEGAHIT (v1.1.3) (Li et al., 2015). This way, the assembly was done across samples combining sequencing reads to improve the reconstruction of genomes even if the sequencing information per sample would have been insufficient, as in the case of samples with low coverage. The assembled contigs were filtered to a minimum length of 2 kb. All sequencing reads were aligned back to the assembled contigs of the co-assembly or the individual samples using BWA-MEM (Li, 2013). The coverage for all contigs was calculated using jgi_summarize_bam_contig_depths in the MetaBAT2 software (Kang et al., 2019). Based on the depth of coverage and nucleotide composition of the contigs, metagenomic binning was performed using MetaBAT2.

To improve the quality of bins, all bins that passed the completeness and contamination filters were aggregated and dereplicated using dRep (Olm et al., 2017). Bins were dereplicated at 99% average nucleotide identity (ANI) to obtain MAGs that were taxonomically equivalent to microbial strains. The quality of the resulting bins was evaluated using CheckM (Parks et al., 2015) with lineage_wf. The completeness and contamination were calculated for all bins, and only those with a completeness ≥80% and contamination ≤10% were used for downstream analyses. The total average coverage for each of the final MAGs was estimated as the mean coverage of all contigs belonging to the same MAG. MAG abundance was estimated as the average read coverage of each bin per sample, correcting for variations in bin lengths and sequencing depth. Furthermore, differential abundance analysis was performed using DESeq2 (v.1.22.1) (Love et al., 2014) to identify MAGs with significant differences in abundance between time points. Significance was determined using the Wald test, *p*-values were adjusted with the Benjamini–Hochberg method, and only hits with a false discovery rate (FDR) <1% and log2 fold change of at least 2 were reported.

The MAGs assembled in this study were compared to two recently available genome collections from the chicken gut microbiome comprising 469 genomes from Glendinning et al. (2020) and 5595 genomes from Gilroy et al. (2021). The Mash genome distances were estimated between the all genomes of each of the datasets to the ones characterized in the current study. A Mash distance of ≤0.05 was used as the species-level threshold (compared to an ANI of ≥95%) (Ondov et al., 2016), i.e., if two genomes had a distance of ≤0.05, then they were considered the same species. Similarly, a Mash distance of 0.34 was used as a genus-level threshold.

### MAG Taxonomic and Functional Annotation

Taxonomic annotation of each representative species was performed with GTDB-Tk v0.3.3 (Chaumeil et al., 2019) using the “classify_wf” function with default parameters. The classification was done against the GTDB database (release 89). MAGs were assigned at the species level if the ANI to the closest GTDB-Tk species representative genome was ≥95%, and the alignment fraction (AF) was ≥65%. A phylogenetic tree was generated with Phylophlan 3.0 (Asnicar et al., 2020) and visualized and annotated with Interactive Tree Of Life (iTOL v5.6.2) (Letunic and Bork, 2019). Protein annotations for identifying enzymes involved in the production of butyrate and propionate and having the ability to remove H_2_ through acetogenesis producing acetate, were obtained by gene prediction and functional annotation with Rapid Annotation using Subsystem Technology (RAST). ORFs were predicted using Prodigal (Hyatt et al., 2010), and the function assignment was performed using a k-mer-based approach with the FIGfam protein family collections from the SEED project (Overbeek et al., 2014). Further, the presence and abundance of selected genes encoding proteins mediating interactions with the host (Medvecky et al., 2018; Rychlik, 2020), including 32 genes involved in flagellar structure assembly were characterized in the MAGs. Given that flagellum assembly requires many proteins and is a very complex process (Apel and Surette, 2008), only genomes carrying at least 70% of these genes are reported here as having the potential to have a flagellum.

CAZymes were annotated on the MAGs using the software dbCAN2 (with options prok-c cluster) (Zhang et al., 2018). The six major CAZyme classes were identified: glycosyltransferases (GTs), glycoside hydrolases (GHs), polysaccharide lyases (PLs), carbohydrate esterases (CEs), carbohydrate-binding module (CBM), and enzymes for auxiliary activities (AAs). For this analysis, all CAZymes commonly identified by two of the three tools used by the dbCAN2 software were taken as positive hits. Because GHs are the most abundant CAZymes, GH families were grouped according to their substrate specificities.

### Statistical Analyses

Principal component analysis (PCA) based on the abundance of each MAG in each sample was used to evaluate the similarity of the sample based on their MAG profile. The significance of the sample groupings observed in the PCA was tested with a PERMANOVA analysis using the function adonis of the R package Vegan (Oksanen et al., 2019). The Shannon and Chao1 alpha diversity indices were estimated based on the MAG contents and differences between the time points were assessed overall with Kruskal–Wallis rank-sum tests, followed by pairwise comparisons with Bonferroni correction to adjust for multiple testing. The association between different bacterial MAGs and CAZymes was inferred from an undirected co-occurrence analysis, using Spearman’s rank correlation coefficients >0.8, with *p* < 0.01. The co-occurrence network was visualized using the igraph package (Csardi and Nepusz, 2006) in R.

## Results

### Assembly of Over 100 Bacterial Genomes From the Chicken GIT

A total of 751 samples were analyzed, generating over 240 giga base pairs (Gb) of sequence data from the gut of 150 birds after trimming and decontamination. The mean number of sequenced reads per sample was 4,270,323, and the total sequencing reads for each sample included in the analyses are shown in Supplementary Table 1. Using the combined data of all samples for the co-assembly, it was possible to obtain bins with lower coverage on individual samples, which would not have been detected in the single-sample binning. Binning the contigs resulted in 421 bins from the single-sample assemblies and 423 bins from the co-assembly. After dereplication and filtering, a total of 155 MAGs were obtained with a high level of completeness (≥80%) and a low level of contamination (≤10%). Specifically, 65.81% of the MAGs had a completeness >90%, and 81.29% of the MAGs had contamination of <5%. MAG completeness and contamination percentages are shown in detail in Supplementary Table 2. From the final set of MAGs, 146 were derived from the co-assembly, supporting pooled sequencing reads to improve genome detection. The MAGs ranged in size from 1.1 to 4.2 megabases (Mb). General MAG characteristics, number of scaffolds per bin, and scaffold N50 are presented in Supplementary Table 2. Compared to the genomes described by Glendinning et al. (2020) and based on the Mash genome distances, 68 out of 155 MAGs had a similar genome at the species level, with 83 MAGs with a comparable genome at the genus level (Supplementary Figure 1) were not close enough to be considered the same species. Additionally, Gilroy et al. (2021) published recently a large set of MAGs from the chicken GIT. The majority of the MAGs assembled in the present study showed a very close genome in the Gilroy dataset, with 115 genomes having a match at the level of species and 39 genomes having a match at the genus level. Only one MAG belonging to the order Christensenellales did not show any corresponding genome in Gilroy dataset (Supplementary Figure 1).

**FIGURE 1:**
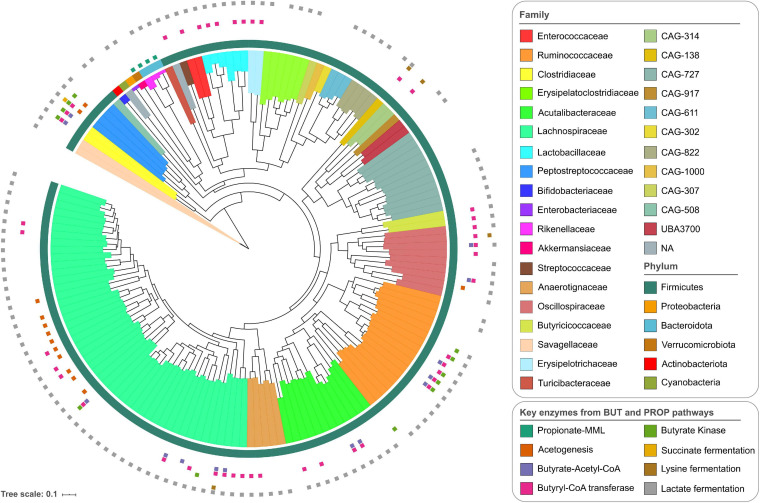
Phylogenetic tree of the 155 metagenomic assembled genomes derived from the chicken gastrointestinal tract (GIT). The outer ring shows the annotations at the phylum level, and the internal coloring reflects the annotation at the family level. The small colored squares outside the phylogenetic tree indicate the presence of all enzymes for the butyrate and propionate production pathways: propionate-MML (methylmalonyl-CoA mutase, methylmalonyl-CoA epimerase, and Methylmalonyl-CoA decarboxylase); acetogenesis (acetyl-CoA synthase corrinoid iron-sulfur protein and 5-methyltetrahydrofolate); butyrate-Acetyl-CoA (3-hydroxybutyryl-CoA dehydrogenase, 3-hydroxybutyryl-CoA dehydratase, butyryl-CoA dehydrogenase, phosphate butyryltransferase); butyryl-CoA transferase; butyrate kinase; succinate fermentation (succinate-semialdehyde dehydrogenase, 4-hydroxybutyrate dehydrogenase, 4-hydroxybutanoyl-CoA dehydratase, succinyl-CoA synthetase); lysine fermentation (lysine 2,3-aminomutase, 3,5-diaminohexanoate dehydrogenase, 3-keto-5-aminohexanoate cleavage enzyme, 3-aminobutyryl-CoA ammonia-lyase); and lactate fermentation (lactate dehydrogenase).

The total average coverage estimated for each MAG based on the pooled data from all samples showed depths of more than 10x for all of them, and in 32 MAGs, a depth of more than 100x (Supplementary Table 2 and Supplementary Figure 2A). The coverage of individual MAGs was uniform across the entire length of the genomes. Two representative MAGs are presented in Supplementary Figures 2B,C, showing changes in coverage over time. The average mapping rate of sequencing reads to MAGs per sample was 3.75%. Based on the taxonomic classification, it was possible to assign 30% of the MAGs to a known species compared with public databases. At higher taxonomic levels, 80% of the MAGs were classified at the genus level, and 98% of the genomes were assigned to a known family (Supplementary Table 3). The MAGs belonged to six different phyla including Firmicutes, Bacteroidota (previously known as Bacteroidetes, but here the GTDB-Tk nomenclature has been used), Verrucomicrobiota, Proteobacteria, Actinobacteriota, and Cyanobacteria, which are common representatives of the chicken GIT microbiome (Xiao et al., 2017; Medvecky et al., 2018; Glendinning et al., 2020). The most common phylum was Firmicutes, which comprised 95% of the MAGs, with genome representatives of 26 families, including Lachnospiraceae (48 MAGs), Ruminococcaceae (17 MAGs), and Acutalibacteraceae (12 MAGs) (both from Oscillospirales) (Figure 1). The most common genera in the Lachnospiraceae family were *Lachnoclostridium*, *Blautia*, and *Faecalicatena*. Furthermore, *Faecalibacterium* and *Eubacterium* were the most common genera in the Ruminococcaceae and Acutalibacteraceae families, respectively. For the other phyla, there were fewer representative families. Differences in the abundance of the MAGs are shown in the heatmap in Supplementary Figure 3A. After the Firmicutes, the Proteobacteria and Verrucomicrobiota had the highest abundance (Supplementary Figure 3B). The 30 most abundant MAGs in all samples belonged to 11 taxonomic families, including Lachnospiraceae, Ruminococcaceae, and Bifidobacteriaceae (Supplementary Figure 3C).

### Bacterial Diversity Increases With Age

**FIGURE 2:**
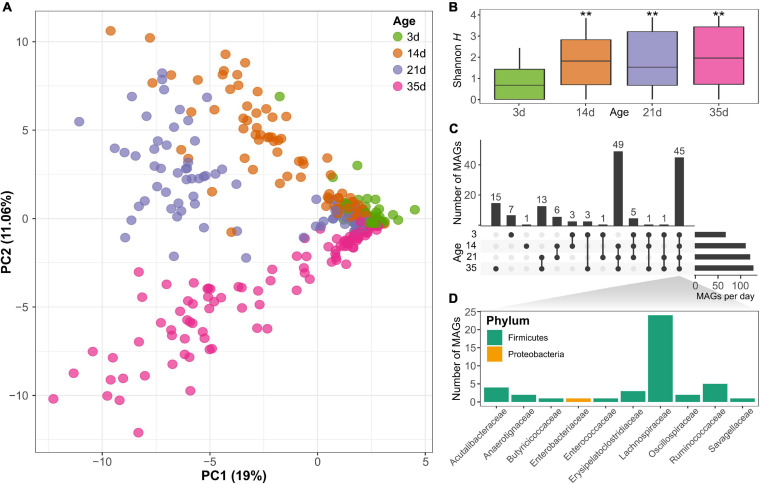
**(A)** Principal component analysis based on the metagenomic assembled genomes (MAGs) content clearly differentiates between samples at different ages (PERMANOVA; *p* < 0.001). **(B)** Shannon diversity increased significantly with the age of the chicken (**Kruskal-Wallis rank-sum test; *p* < 0.001). **(C)** Number of MAGs shared at different time points reveals there is a core group of 45 bacterial genomes that can be identified at all time points. **(D)** The core genomes present at all time points belong to several families mainly of the phylum Firmicutes, especially Lachnospiraceae.

Principal component analysis of MAG abundance showed a significant separation of the samples according to the age of the animals (PERMANOVA; *p* < 0.001), visible especially for the samples after day 3 based on the first two principal components that explained 44% of the variance (Figure 2A), indicating the change in the microbiome composition of the chickens over time. In total, there were fewer MAGs present on day 3 than at any of the subsequent sampling points. This is also reflected in a significant increase in MAG diversity at day 14 (Kruskal–Wallis rank-sum test; *p* < 0.001), as shown by the Shannon diversity index (Figure 2B). No significant differences in Shannon diversity were observed after day 14 (between day 14 and 21; nor between day 21 and 35), showing a stabilization in diversity after the first 2 weeks of age. Based on the Chao1 index, which gives more weight to rare species, there were also significant differences between the time points (Kruskal–Wallis rank-sum test; *p* < 0.001) and an increase not only at day 14, but also at day 35 (Supplementary Figure 4). Considering the MAG abundance and distribution across samples, 45 MAGs were present at all time points sampled, of which most belonged to the Lachnospiraceae family of the Firmicutes phylum, representing a set of core organisms that colonized the gut from early developmental stages and were present throughout the sampling time points (Figures 2C,D). On the other hand, there were also genomes identified only at specific time points. For example, 49 MAGs were identified only in chickens on day 14 and older. Furthermore, 15 genomes were detected only at the latest time points sampled on day 35 (Figure 2C). The relative abundances of the MAGs are displayed in Supplementary Figure 3 and show differences in the composition of the microbiome at the four time points evaluated. Differential abundance analysis revealed a significant change (log2 fold change >2 or <−2 and FDR < 1%) in the abundance of 71 MAGs between day 35 and day 3, 26 MAGs had lower abundance and 45 MAGs had higher abundance at day 35 than on day 3 (Supplementary Figure 5). Of the MAGs showing a reduced abundance at day 35, the majority were from the Lachnospiraceae family. More families were represented in the MAGs that increased in abundance, among which several representatives of the Lachnospiraceae family were identified. This shows that several family members could be found at all time points but that their abundance changes with the age of the chickens. Other MAGs from families from the class Clostridia, such as Oscillospiraceae, also showed a significant increase in abundance at day 35 (Supplementary Figure 5 and Supplementary Table 4).

### Butyrate and Propionate Production and Acetogenesis Capacity Are Distributed in Different Species

To investigate the ability of the identified bacteria to produce SCFAs, particularly propionate and butyrate, the presence of the genes required to produce these compounds was investigated. Genes required for butyrate production (from acetyl-CoA through the classical pathway via butyrate kinase or from indirect routes through the conversion of acetate, succinate, or lactate) were generally more common in the Firmicutes phylum (Figure 1 and Supplementary Figure 6), particularly in *Faecalibacterium* and *Gemmiger* (Ruminococcaceae) and *Clostridium* (Clostridiaceae). Several other genomes have butyryl-CoA transferase, the most common enzyme of the butyrate-producing pathway and was found in 44 genomes. Butyrate kinase was mainly found in Ruminococcaceae. Other genes involved in the production of butyrate from alternative pathways were less commonly found in the MAGs, for example, in the genomes of Peptostreptococcaceae, Lachnospiraceae, and Oscillospiraceae. For the propionate production (via the succinate pathway by the conversion of succinate to methylmalonyl-CoA), enzymes for the succinate-methylmalonate pathway, encoded by genes for methylmalonyl-CoA mutase, epimerase, and decarboxylase, were found in all the genomes of the Bacteroidota and Verrucomicrobiota phyla. Complete enzyme sets for propionate production were not found in any of the other MAGs described here.

Acetogenesis capacity was also evaluated in the MAGs, given that this process is important for the production of acetate and the removal of H_2_ which is produced during carbohydrate metabolism (Carbonero et al., 2012; Medvecky et al., 2018). The potential to remove H_2_ through acetogenesis was identified in the genomes of mainly Lachnospiraceae strains, and in two genomes of the Peptostreptococcaceae family, all of which carry the genes for the corrinoid iron-sulfur acetyl-CoA synthase and the 5-methyltetrahydrofolate methyltransferase.

### A Diverse Collection of Carbohydrate-Degrading Genes Is Present in the Chicken Microbiome

**FIGURE 3:**
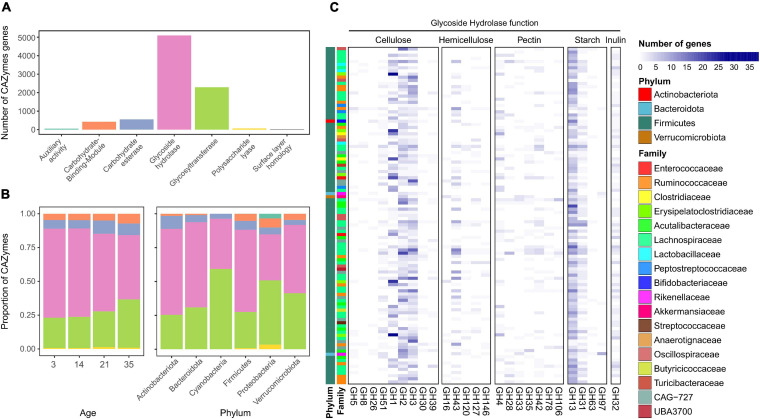
**(A)** Number of Carbohydrate Active Enzymes (CAZymes) genes identified in the metagenomic assembled genomes (MAGs) from the chicken gut. **(B)** Proportion of CAZymes at different ages and in the different taxonomic families. **(C)** Abundance and distribution of the most commonly found CAZyme genes from the glycoside hydrolase family according to MAG family and phylum.

More than 8,000 CAZymes were identified in the 155 MAGs (Supplementary Table 5) with the most common classes belongs to glycoside hydrolases (GHs), with 5,092 genes identified (Figure 3A). The next most abundant category corresponded to glycosyltransferases (GTs). The two lowest represented categories were enzymes with AAs and PLs. There were differences in the proportions of these categories present in chickens of different ages (Figure 3B). At earlier stages, the microbiota was dominated by GHs, which decreased over time. On day 35, there was a higher proportion of GTs (χ^2^ test; *p* < 0.05) and a decreased proportion of GHs (χ^2^ test; *p* < 0.001). Firmicutes and Bacteroidota showed a high proportion of GHs (Figure 3B), whereas Proteobacteria and Cyanobacteria showed a higher proportion of GTs. Proteobacteria also had a higher proportion of AAs and PLs than the other genomes. The most commonly identified GH was the GH13 family (Figure 3C), which combines many enzymes responsible for the degradation of starch (Stam et al., 2006; El Kaoutari et al., 2013). The following most common families found in the chicken MAGs were GH1, GH2, and GH3, which are mostly related to cellulose degradation. Additional GHs identified in other genomes were involved in hemicellulose, pectin, and inulin utilization.

### Several Proteins Important for the Bacterial Interaction With the Host Were Identified

Multiple bacterial interactions with their hosts were assessed by identifying genes, such as glycosaminoglycans (GAGs), including heparin, chondroitin sulfate, and hyaluronan, which are essential components of the extracellular matrix in animals (Kawai et al., 2018). Several bacteria target these GAGs for adherence and colonization of the host cells and carry enzymes capable of breaking down GAGs (Kawai et al., 2018). All identified genes are shown in Supplementary Table 6. A gene coding for a chondroitinase was identified in one Enterobacteriaceae genome, and a gene for heparinase was found in the Acutalibacteraceae genome. No genomes carrying genes encoding hyaluronidase or collagenase were found. Furthermore, hemagglutinin and hemolysin expression also impact the adherence and lysis of the host cells; hence, their activities have been associated with pathogenic bacteria (Goebel et al., 1988; Clantin et al., 2004). These genes were identified in three Rikenellaceae MAGs but none of the Firmicutes genomes. One of these genomes also carries a mucin-desulfating sulfatase gene, which is involved in the degradation of mucins (Tailford et al., 2015; Medvecky et al., 2018). Genes for glutamate decarboxylase (GAD) were found in the genomes of the phyla Bacteroidota, Proteobacteria, Firmicutes, and Verrucomicrobiota. A full set of proteins related to flagella assembly, which play significant roles in bacteria-host interactions by allowing attachment and invasion (Rossez et al., 2015), was identified in at least six MAGs from the Firmicutes phylum and one from the Proteobacteria phylum.

### Co-occurrence of Bacterial Groups and CAZymes Identified Key Components of the Community

**FIGURE 4:**
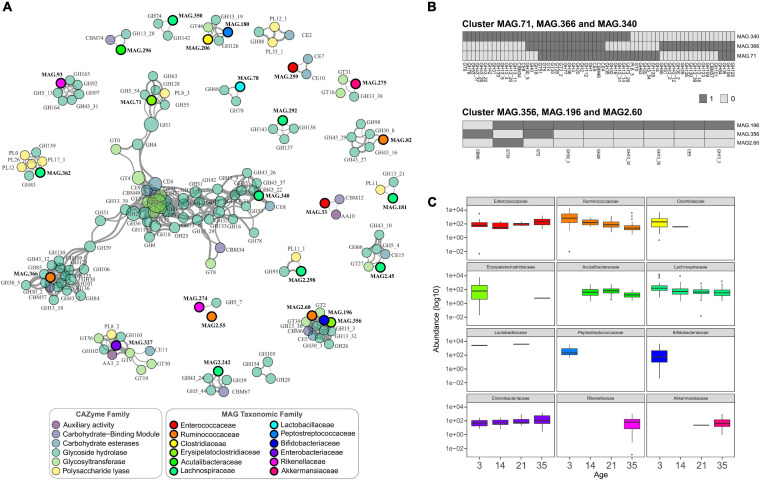
**(A)** Co-occurrence network of main CAZymes genes and main taxonomic families. Nodes represent CAZymes genes (colored by CAZyme family) or phylogenetic families, and their size represents their abundance. The edges thickness is proportional to the Spearman’s rank correlation between the nodes; only strong and significant correlations are shown (Spearman’s rank coefficient >0.8, *p* < 0.01). **(B)** Heatmaps of the presence and absence of CAZymes in the MAGs from two of the main clusters shown in **(A)**. **(C)** Abundance of the taxonomic families of the MAGs represented in the co-occurrence network. The boxplots are colored based on taxonomic families following the same scheme as shown in **(A)**.

To study how the MAGs interact in the microbial community as a whole and to generate insights into the potential mechanisms of these interactions, a co-occurrence analysis was performed between all the recovered genomes and the complete set of CAZymes identified. A total of 564 significant positive correlations between CAZyme families and MAGs were identified, with several connected clusters or modules (Figure 4A). Negative correlations were not higher that 0.8 and did not show significance values (not shown). The largest module corresponded mainly to CAZymes from GT2_2, and GH3 families showing that independent of the genome location, it was possible to distinguish groups of CAZymes that co-occur in the GIT samples (Figure 4A). These two enzyme families had the highest closeness centrality (the closeness of other nodes) and high node degree (number of connections), highlighting that these are key enzymes in the network because of their high interconnection with other CAZymes and bacteria. Additionally, these enzymes were identified in many bacteria across different taxonomic groups, with GT2_2 being present in 150 genomes and GH3 in 122, underscoring their importance (Supplementary Table 7). At the bacterial level, three genomes of the genera *Faecalicatena* (MAG.340), *Erysipelatoclostridium* (MAG.71), and one from the Ruminococcaceae family (MAG.366) showed high co-occurrence and high node degree, suggesting that they represent potential keystone species (Berry and Widder, 2014) serving as connection centers in the network. Given their position and connectivity, these bacteria represented interaction hubs, and it is expected that changes in their occurrence can lead to major changes in the GIT microbiome.

Other associations were observed between MAG2.60 (*Fournierella*), MAG.196 (*Bifidobacterium*), and MAG.356 (*Erysipelatoclostridium*), simultaneously being strongly correlated with the CAZymes of the GH family, two GTs, a CBM, and a CE. The presence/absence of the CAZymes in individual MAGs present in the same clusters revealed that many enzymes were not located within the same genome but were present in other genomes (Figure 4B). As shown in the heatmaps in Figure 4B, some enzymes were present in the genomes of the three species, including the very common GH1, GH2, and GH3. Additionally, several enzymes were mainly contributed by only one species in this cluster, with MAG.340 (*Faecalicatena*), for example, carrying most of the GH13 and GH43 starch and hemicellulose degrading enzymes, respectively.

CAZymes enable the utilization of a great variety of carbon sources in the GIT (El Kaoutari et al., 2013), their presence and abundance at specific developmental time points can also impact the bacterial changes. The abundance of particular bacterial families, such as Ruminococcaceae, showed a reduction over time, with a significantly lower abundance at day 35 compared to day 3 (Wald test; *p* < 0.001) (Figure 4C). From the co-occurrence analysis, the Ruminococcaceae MAGs were highly correlated with many GHs. In the case of the cluster of *Fournierella, Bifidobacterium*, and *Erysipelatoclostridium*, all these bacteria were more abundant at the first sampling time point, indicating that their associated CAZymes were also more abundant in the earlier stages. As shown in Figure 4C, MAGs that showed significant CAZyme associations from the Bifidobacteriaceae and Peptostreptococcaceae were identified only in young chickens, at day 3. On the other hand, families detected at later time points, such as Rikenellaceae and Akkermansiaceae, which points to an increase in the CAZymes highly correlated with them in the older chicken.

## Discussion

In this study, 155 draft microbial genomes were reconstructed through metagenomic sequencing and binning to expand the current understanding of broiler chicken gut microbiota and their nutritional function. The assembled genomes represent a smaller number compared to recently published MAG sets derived from the chicken GIT (Glendinning et al., 2020; Gilroy et al., 2021). The shallower sequencing depth per sample analyzed in this study was the main determinant for the number of genomes assembled. The current study complements previous data sets by focusing on the functional characterization of the genomes, adding new information about the complexity of the chicken gut microbiome, the carbohydrate metabolism potential of these bacterial genomes and their dynamic changes in abundance over time.

The six phyla reported here (Firmicutes, Proteobacteria, Bacteroidota, Verrucomicrobiota, Actinobacteriota, and Cyanobacteria) have also been identified previously by different experimental approaches (Xiao et al., 2017; Huang et al., 2018; Medvecky et al., 2018; Glendinning et al., 2020). The study by Gilroy et al. (2021) comprising a very extensive taxonomic classification of MAGs in the chicken microbiome, also revealed a similar taxonomic composition as the one shown with the here, with a dominance of the phyla Firmicutes and Bacteroidota. The increase in diversity and subsequent stabilization based on the 155 MAGs, especially when looking at the Chao1 index that gives more importance to less common species, closely reflects similar trends observed in previous studies. Based on 16S rRNA amplicon sequencing, Oakley and Kogut (2016) and Jurburg et al. (2019) observed a similar increase in microbiome diversity at 3 and 6 weeks post-hatch and within the first 35 days, respectively. These variations might be attributed to developmental changes in the chicken GIT, changes in the diet, and even to the development of the immune system. Proteobacteria representatives colonize the cecum in the early stages of development followed by the rapid-growing Lachnospiraceae and Ruminococcaceae families, as observed at high abundance at approximately day 3 (Jurburg et al., 2019; Kubasova et al., 2019). At later time points, representatives of the phylum Bacteroidota appeared in the GIT with slower-growing and more specialized taxa, such as *Alistipes* (Rikenellaceae) and *Akkermansia* (Akkermansiaceae) (Huang et al., 2018; Jurburg et al., 2019), which carry genes for specific SCFA production.

The presence of Firmicutes, particularly the orders Lachnospirales and Oscillospirales (class Clostridia) in the chicken gut has been attributed to environmental sources compared to other bacteria from Bacteroidota and Actinobacteriota, which are more efficiently transferred from adult hens to newly hatched chickens (Kubasova et al., 2019). Clostridia comprise a substantial part of the GIT microbiota involved in the maintenance of gut function (Lopetuso et al., 2013). Even though bacterial genomes of this class were identified at all time points, we showed that there were significant changes in the composition of the gut microbiome over time. These dynamic changes in the GIT have an impact on the metabolic potential of the microbiome. For instance, Firmicutes was the phylum with a larger number of taxa encoding enzymes required for butyrate production. In particular, the Ruminococcaceae and Clostridiaceae families carrying genes from the butyrate pathway, are known to be among the first bacteria to colonize the ceca of newly hatched chickens (Onrust et al., 2015; Polansky et al., 2016). Commensal Clostridia colonize the intestine in the early stages and participate in the modulation of physiological, metabolic, and immune processes, in part through the release of butyrate as an end-product of fermentation (Pryde, 2002; Lopetuso et al., 2013). Butyrate is then used as the main energy source for enterocytes and is involved in many biological processes (Guilloteau et al., 2010; Koh et al., 2016; Beauclercq et al., 2018). Therefore, identification of strains carrying the enzymes necessary for butyrate production present from the early stages of development, such as *Faecalibacterium* and *Clostridium* presented in this study, is an important step toward future intervention and modulation of the gut microbiota to improve the overall health and growth performance of poultry.

On the other hand, propionate is a less preferred substrate of colonocytes but is transported to the liver and it is an important energy source for the host (Koh et al., 2016). The presence of bacteria of the Rikenellaceae and Akkermansiaceae families, carrying genes for propionate production, agrees with the reported increase in propionate concentrations in broiler chickens from undetectable levels on day 1 to high and stable concentrations from day 14 (van der Wielen et al., 2000; Guilloteau et al., 2010). Representatives of the phylum Bacteroidota are among the main propionate-producing bacteria in the chicken GIT (Polansky et al., 2016). The expression of enzymes for propionate metabolism dominates in this phylum, as observed for the species in this study.

Furthermore, the presence of genes required for reductive acetogenesis in the Lachnospiraceae family, has also been reported in other studies (Sergeant et al., 2014; Medvecky et al., 2018). Their general distribution over the entire time frame studied shows the importance of the constant removal of H_2_, which is a typical by-product of the fermentation of carbohydrates, and a high concentration of H_2_ can inhibit glycolysis. Genes for methanogenesis, another pathway for H_2_ removal, were not identified in the MAGs.

Medvecky et al. (2018) characterized the functions of specific isolates from the chicken cecum and observed the utilization of different strategies for gut colonization. Genes important for adherence to host cells, such as hemagglutinin, were found mainly in Bacteroidota genomes, similar to what we report in the present study. The GAD gene catalyzes the production of γ-aminobutyrate (GABA) from glutamate, which plays multiple physiological functions both for the host and for bacteria (Strandwitz et al., 2019), and increases resistance to highly acidic environments, providing advantages to survive in the stomach (Feehily and Karatzas, 2013). Furthermore, several Firmicutes bacteria carry genes for flagellar assembly and motility, but no Bacteroidota with these machinery genes, in line with previous genomes isolated from the chicken cecum (Medvecky et al., 2018).

Regarding CAZyme-encoding genes, GHs constitute some of the main enzymes found in the microbiome of a wide range of species (El Kaoutari et al., 2013; Jose et al., 2017; He et al., 2019; Glendinning et al., 2020). GHs catalyze the hydrolysis of glycosidic bonds found in complex carbohydrates, including starch. As starch from the cornmeal was the main source of carbohydrates in the diet of the chicken in the current study, it was not surprising to see a high potential for digestion, with many genes encoding GHs. The high abundance observed for the GH13 family underlines its importance, comprising the main group of enzymes that digest resistant starch that reaches the colon and cecum (Stam et al., 2006; Zhang et al., 2020). Furthermore, due to the importance of the GH3 and GT2 families in the network, variations in these enzymes, and thereby of the metabolites produced from the degradation of specific carbohydrates by these enzymes, may have a stronger influence also on the microbiome community. The GH3 family includes widely distributed enzymes with diverse activities such as cellulose and bacterial cell wall degradation (Harvey et al., 2000; Faure, 2002). This, together with their high ubiquity, supports their central role in the microbiome community.

To date, scarce information is available on the changes in the abundance of these enzymes in chicken GIT over time. Different CAZyme profiles have been shown across various age groups in the human gut microbiome, with distinct taxonomic drivers of these profiles (Bhattacharya et al., 2015). With the increase in diversity observed over time, the overall carbohydrate metabolism capacity also increased, allowing the microbiome to digest a wider range of carbohydrate substrates as the chicken transitions to the adult stage.

The co-occurrence network analysis revealed significant correlations between specific bacteria and groups of CAZymes and helped to better resolve the abilities of these microorganisms with regard to carbohydrate metabolism. The correlations found could be derived from biologically relevant symbioses between different species carrying the corresponding enzymes and in other cases, the CAZyme-MAG associations corresponded to enzymes in a single species encoded in the same genome. The presence and absence of enzymes in the bacteria of the main clusters of the network indicate that there is a division in the ability to digest different carbohydrates. The genus *Faecalicatena*, carrying many CAZymes in the network, is not very well studied, and multiple species have been reclassified as members of this novel genus (Sakamoto et al., 2017). However, its central role in the network suggests that further studies are required. Recently, Xiao et al. (2020) compared the gut microbiome in different bird species and found that birds fed a corn-soybean basal diet showed a strongly interconnected microbial community with species from the classes Clostridia and Bacteroidia. In line with these findings, although the 155 genomes presented in this study constitute only a small sample of the GIT microorganisms, there was also a strong representation of bacteria in these classes in the network, with bacteria genera *Alistipes*, *Ruminococcus*, *Fournierella*, and *Faecalicatena*.

Differences observed in the taxonomic composition and CAZyme abundance over time provided insights into the dynamics leading to microbial succession. They also highlighted the importance of considering the time point variation in the development and administration of probiotics to modify chicken gut health. The division in the enzymes present in different bacterial genomes points to a system where each microorganism can degrade a certain set of complex carbohydrates depending on the machinery it carries. Compositional changes would lead to differences in the capacity of the microbial community to digest complex carbohydrates. With additional studies of CAZyme changes under different conditions, these enzymes could become useful biomarkers of the functional diversity and carbohydrate potential of the gut microbiome without focusing on single isolated bacteria that are not a real representation of the community.

A limitation of the present study is that even though a large number of samples (751) were sequenced and comprehensively analyzed, some reached lower sequencing depths than others, making single-sample assemblies difficult. By pooling the sequencing data together, higher quality assemblies were possible, especially for bacterial strains present in multiple samples. Yet, underestimation of low prevalent/low abundance species is possible. Our study used a genome-centric approach to obtain complete or nearly complete genomes. Other analyses, like gene-centric approaches could increase the number of sequencing reads mapped to genetic components, but would lose the ability to identify which features occur in which specific genomes or taxa. Furthermore, we identified and described the presence of genetic components responsible for SCFA production, but future studies will be needed to validate these results experimentally, e.g., by measuring SCFA metabolite in parallel to the microbiome analysis.

Besides expanding the chicken gut microbial genome repertoire for future studies, these findings substantially advance our understanding of microbiota-associated metabolic pathways, providing new opportunities for improving the overall performance and health of poultry.

## Data Availability Statement

The primary data supporting the findings of this study are available in the Supplementary Material and the NCBI BioProject, ID PRJNA715658.

## Ethics Statement

The animal study was reviewed and approved by the Lower Austrian Region Government, Group of Agriculture and Forestry (approval code LF1-TVG-57/005-2018), and all experiments were performed following the European Guidelines for the Care and Use of Animals for Research Purposes (European Council, 2010).

## Author Contributions

MG, VK, and AK designed the project. MG, NG, and AK performed the experiments. MG and VK supervised the experiments. MS-W performed bioinformatics analysis. MS-W and MG interpreted the data and wrote the manuscript. All authors have read and approved the final manuscript.

## Conflict of Interest

All authors are employed by BIOMIN Holding GmbH (BIOMIN is part of DSM).

## Publisher’s Note

All claims expressed in this article are solely those of the authors and do not necessarily represent those of their affiliated organizations, or those of the publisher, the editors and the reviewers. Any product that may be evaluated in this article, or claim that may be made by its manufacturer, is not guaranteed or endorsed by the publisher.

## Supplementary Material

Supplementary Figure 1 **(A)** Pairwise Mash distance comparison between the MAGs described in this study and the ones published by Glendinning et al. (2020). **(B)** Number of genomes that have a similar species or genus in the published dataset from Glendinning et al. (2020). **(C)** Pairwise Mash distance comparison between the MAGs described in this study and the ones published by Gilroy et al. (2021). **(D)** Number of genomes that have a similar species or genus in the published dataset from Gilroy et al. (2021).

Supplementary Figure 2 **(A)** Total average depth for each MAG over all samples. **(B,C)** The coverage over the total length of two example MAGs per time point. In MAG.227 there is an increase in coverage with the age of the animals, whereas in the MAG.366 a decrease in coverage is observed.

Supplementary Figure 3 **(A)** Abundance of all MAGs according to age of the chicken in days. **(B)** Relative abundance of phyla among the 155 MAGs from the chicken GIT. **(C)** Relative abundance of the 30 most abundant MAGs (colored by taxonomic family) in all the chicken gut samples per day.

Supplementary Figure 4 Chao1 diversity indices based on the MAG composition per sample at each time point (^****^*p* < 0.001; ns, non-significant).

Supplementary Figure 5 Significant differentially abundant MAGs between day 35 and day 3. All MAGs shown have an FDR < 1% and log2 fold change >2 or <−2. The taxonomic family of each MAG is also shown.

Supplementary Figure 6 Number of enzyme genes per MAG identified belonging to the butyrate, propionate, and acetogenesis pathways.

Supplementary Table 1 Metagenomic sequenced samples and total sequencing reads (after quality filtering and removal of host reads).

Supplementary Table 2 General characteristics of MAGs, including completeness and contamination percentage based on CheckM, genome length, number of scaffolds, and scaffold N50.

Supplementary Table 3 Taxonomic assignments for the MAGs.

Supplementary Table 4 Log2 fold changes and adjusted *p* values for MAGs with significant differential abundance between day 35 and 3.

Supplementary Table 5 Total number of CAZymes by category identified in each genome and the percentage of CAZymes in comparison to the total predicted proteins for each MAG.

Supplementary Table 6 Proteins present in the MAGs related to interaction with the host.

Supplementary Table 7 Prevalence of CAZyme genes in each MAG.
