# Supplementary material for: Genome-Resolved Metagenomics of the Chicken Gut Microbiome
Source: Front Microbiol. 2021 Aug 16;12:726923. doi: 10.3389/fmicb.2021.726923 (PMC8415551; doi:10.3389/fmicb.2021.726923)
Supplement: Supplementary Figure 1 — (A) Pairwise Mash distance comparison between the MAGs described in this study and the ones published by Glendinning et al. (2020). (B) Number of genomes that have a similar species or genus in the published dataset from Glendinning et al. (2020). (C) Pairwise Mash distance comparison between the MAGs described in this study and the ones published by Gilroy et al. (2021). (D) Number of genomes that have a similar species or genus in the published dataset from Gilroy et al. (2021). [file Data_Sheet_1.DOCX]

Supplementary Material

**Supplementary Figure 1. (A)** Pairwise Mash distance comparison between the MAGs described in this study and the ones published by Glendinning *et al.* (2020). **(B)** Number of genomes that have a similar species or genus in the published dataset from Glendinning *et al.* (2020). (**C**) Pairwise Mash distance comparison between the MAGs described in this study and the ones published by Gilroy *et al.* (2021). **(D)** Number of genomes that have a similar species or genus in the published dataset from Gilroy *et al.* (2021).

**Supplementary Figure 2. (A)** Total average depth for each MAG over all samples. **(B)** and **(C)** The coverage over the total length of two example MAGs per time point. In MAG.227 there is an increase in coverage with the age of the animals, whereas in the MAG.366 a decrease in coverage is observed.


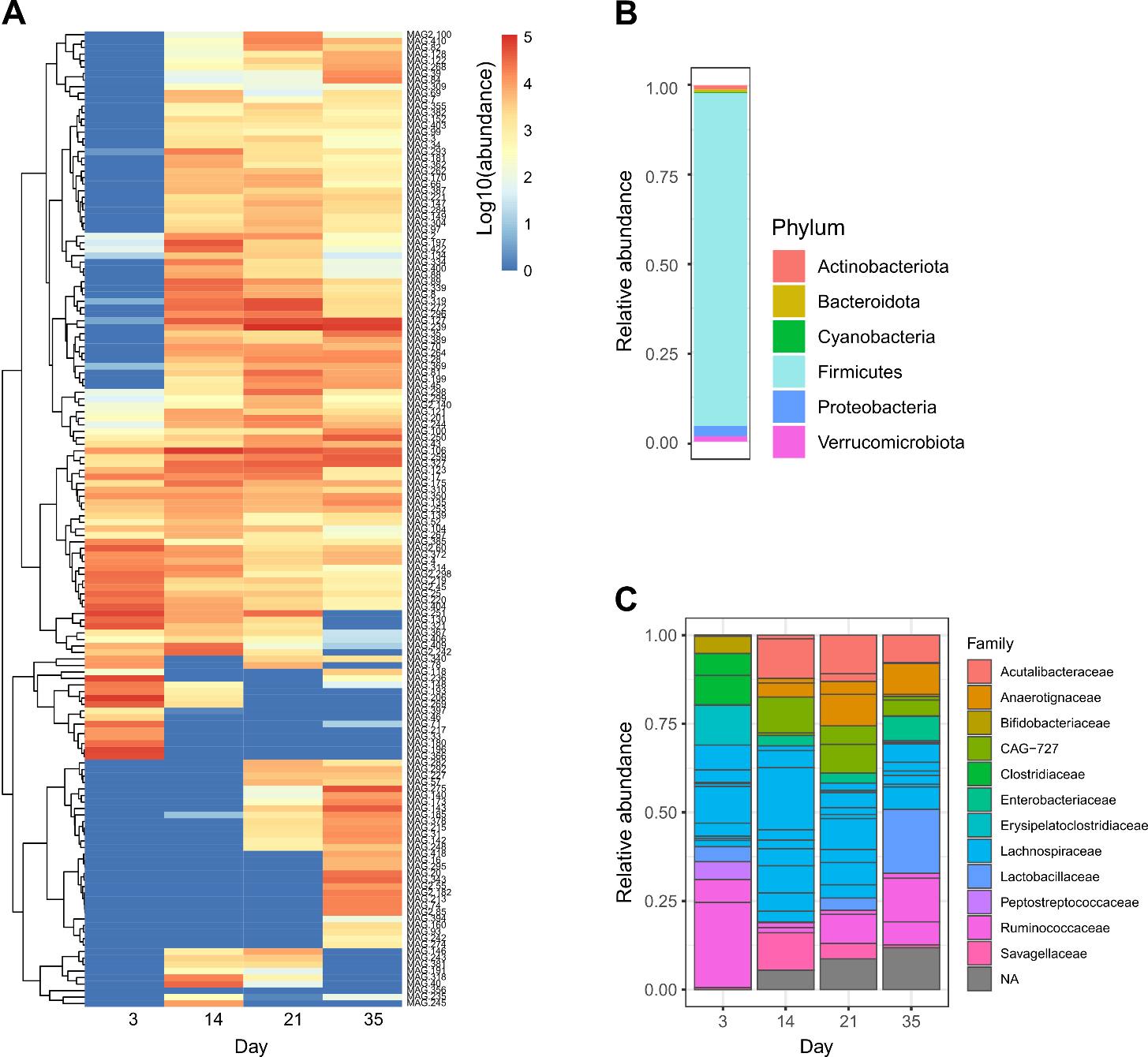


**Supplementary Figure 3. (A)** Abundance (log10-transformed) of all MAGs according to age of the chicken in days. **(B)** Relative abundance of phyla among the 155 MAGs from the chicken GIT. **(C)** Relative abundance of the 30 most abundant MAGs (colored by taxonomic family) in all the chicken gut samples per day.


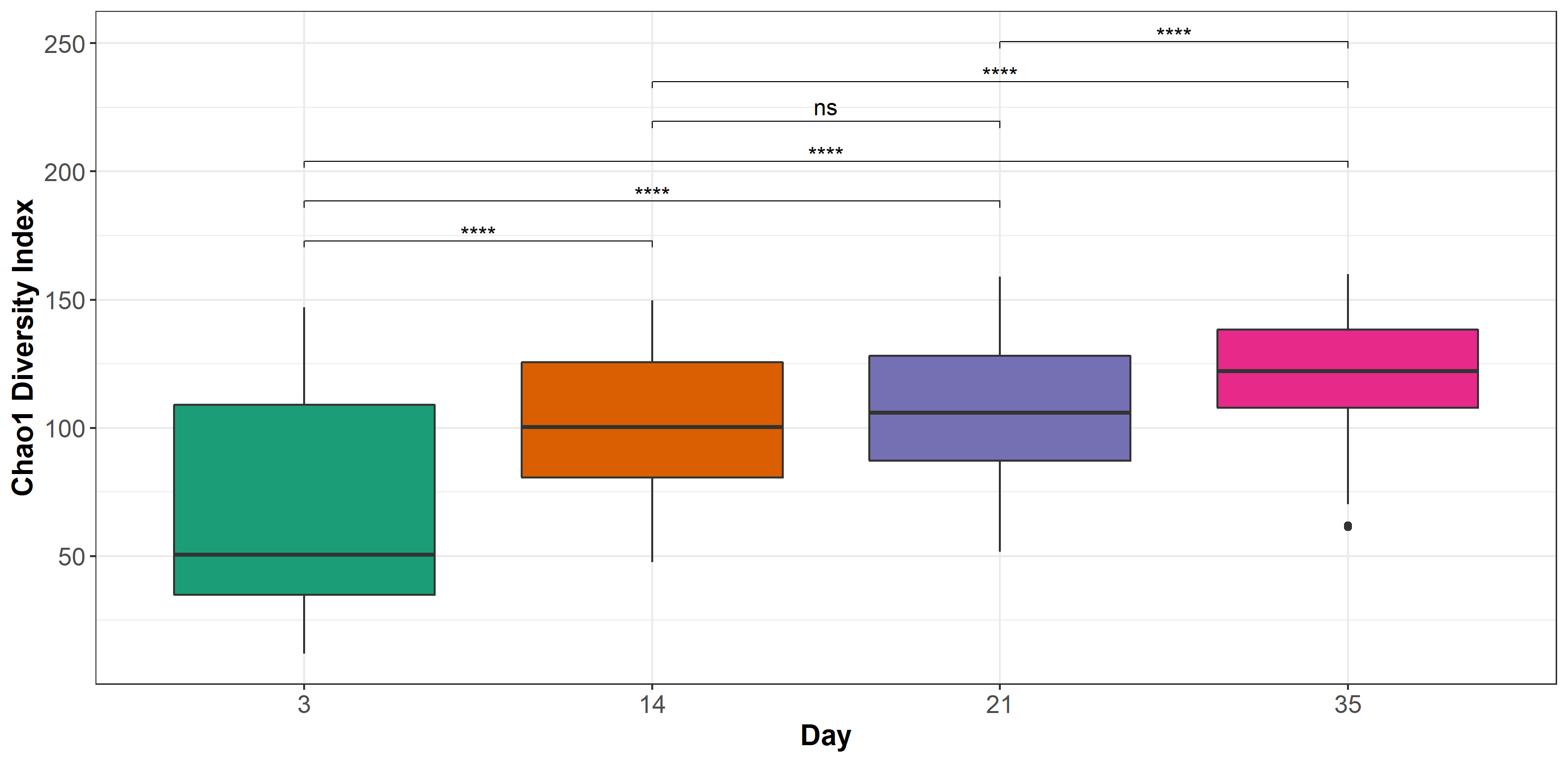


**Supplementary Figure 4.** Chao1 diversity indices based on the MAG composition per sample at each time point (**** = *p* < 0.001; ns = non-significant).


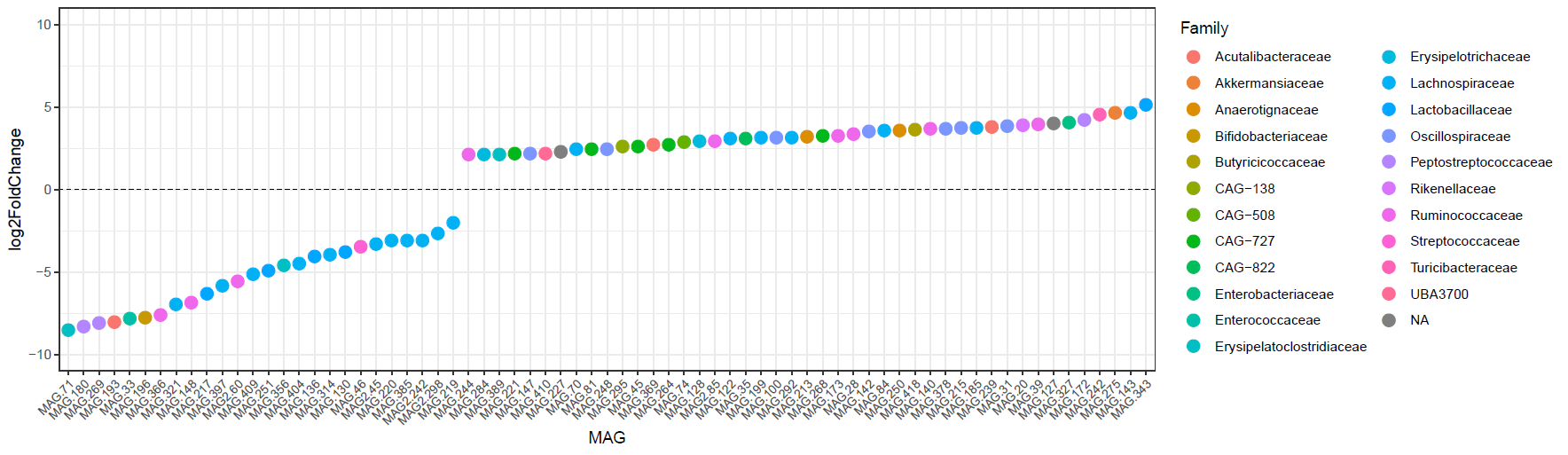


**Supplementary Figure 5.** Significant differentially abundant MAGs between day 35 and day 3. All MAGs shown have an FDR<1% and log2FoldChange >2 or <-2. The taxonomic family of each MAG is also shown. Adjusted p-values and log2FoldChanges are also included in Supplementary Table 4.


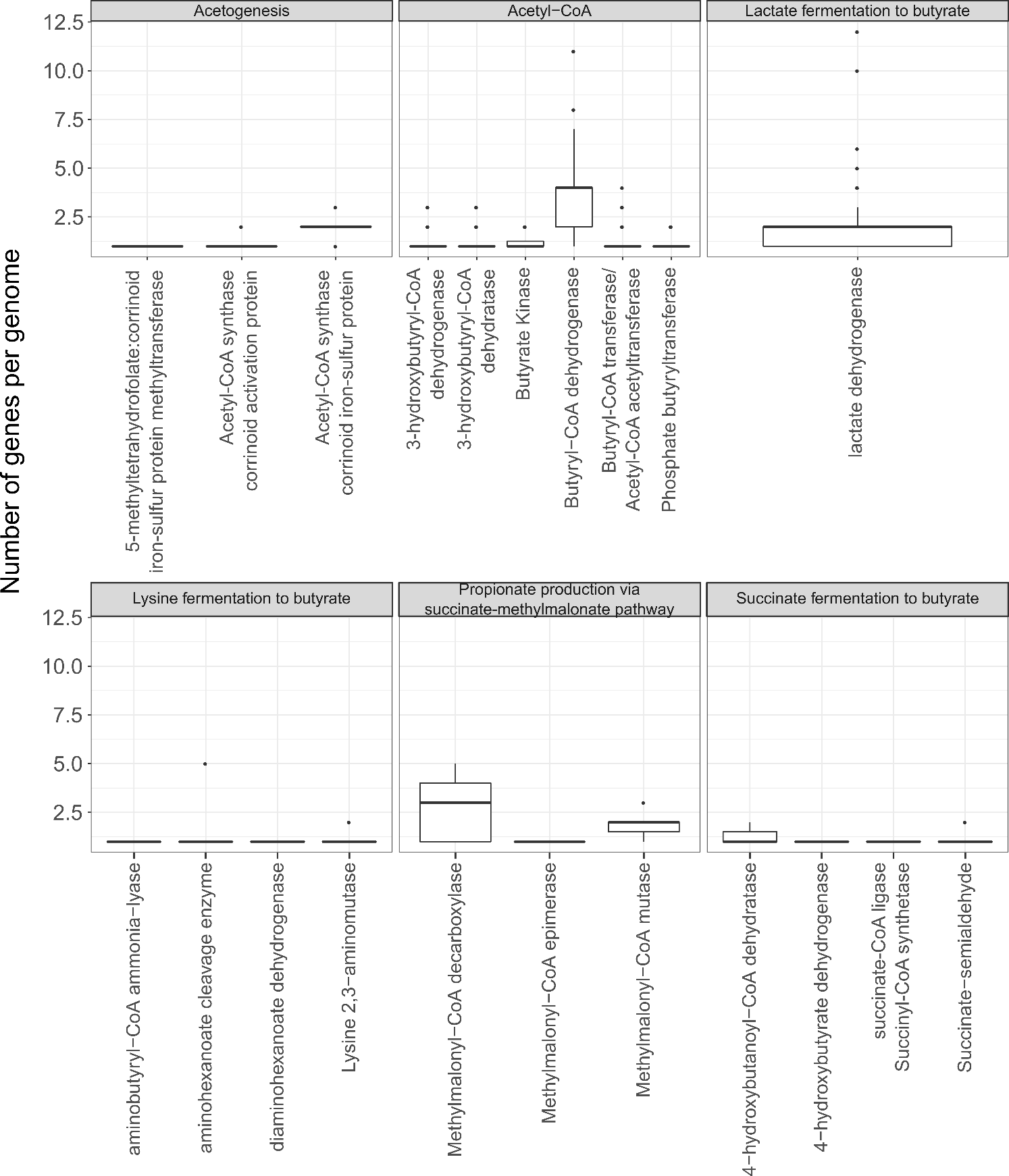


**Supplementary Figure 6**. Number of enzyme genes per MAG identified belonging to the butyrate, propionate and acetogenesis pathways.
